# The relationship between axial length and anterior segment biometry in high axial myopic Chinese students aged 7 to 18 years

**DOI:** 10.3389/fmed.2025.1671451

**Published:** 2026-01-13

**Authors:** Zengrui Zhang, Jingyu Mu, Yanrong Yang, Yun Dai, Junguo Duan

**Affiliations:** 1 Chengdu University of TCM, Chengdu, Sichuan, China; 2 Eye School of Chengdu University of TCM, Chengdu, Sichuan, China; 3 Ineye Hospital of Chengdu University of TCM, Chengdu, Sichuan, China; 4 Key Laboratory of Sichuan Province Ophthalmopathy Prevention & Cure and Visual Function Protection with TCM, Chengdu, Sichuan, China; 5 Retinal Image Technology and Chronic Vascular Disease Prevention & Control and Collaborative Innovation Center, Chengdu, Sichuan, China

**Keywords:** anterior segment biometry, axial length, children, crystalline lens, high axial myopia

## Abstract

**Purpose:**

This study aimed to examine the relationship between axial length (AL) and anterior segment biometry in Chinese children and adolescents with high axial myopia.

**Methods:**

A cross-sectional study was conducted involving 366,278 Chinese students aged 7 to 18 years from January 2020 to December 2022. The SUOER *μ*-Meter optical biometric was used for measuring ocular biometric parameters. A multivariate linear regression model was used to examine the correlation with AL.

**Results:**

Among the participants, 28,877 exhibited high axial myopia (AL ≥ 26 mm). The average keratometry (K) and lens thickness (LT) in the high axial myopia group were significantly lower than those in both the non-myopia and normal myopia groups (all *p* < 0.001). Central corneal thickness (CCT) and anterior chamber depth (ACD) were significantly greater in the high axial myopia group compared to both non-myopia and normal myopia groups (all *p* < 0.001). Multivariate linear regression analysis demonstrated that K (*β* = −0.265), CCT (*β* = 0.001), ACD (*β* = 1.609), and LT (*β* = −0.607) were all correlated with AL (all *p* < 0.001). After adjusting for gender and age, when AL was <27 mm, AL was negatively correlated with LT; similarly, when AL exceeded 27 mm, there was no linear correlation between AL and LT (*p* > 0.05).

**Conclusion:**

In Chinese children and adolescents, a high AL (i.e., ≥26 mm) was associated with significantly lower K, thicker CCT, and deeper ACD, and thinner LT compared with subjects having AL < 26 mm. When AL was <27 mm, LT thinning may contribute to myopia compensation; however, when AL exceeds 27 mm, this compensatory effect of crystalline lens thickness appears to diminish.

## 1 Introduction

The increase in near-work activities and reduced outdoor time, coupled with genetic factors, has contributed to the early onset and high incidence of myopia among young people (1–3). Particularly in East and Southeast Asia, the incidence of myopia among young individuals reaches 80–90%, with rates among school-age children as high as 60% (measured under cycloplegic refraction), far exceeding the rates in Europe (40%) and North America (42%) (2, 4). Progression of myopia can result in serious complications and is a leading cause of irreversible visual impairment and blindness worldwide. It is further associated with increased healthcare expenditures, reduced productivity, and diminished quality of life (5, 6). It is currently established that myopia, especially high myopia, is significantly associated with severe complications, including retinal detachment, retinal neovascularization, early cataracts, and glaucoma (7).

During normal eye development, the emmetropization process coordinates the growth of axial length (AL) with the optical power of the cornea and lens to achieve optimal focus (8). This process involves complex interactions between anterior segment structures and ocular elongation. Disruption of this coordination leads to excessive AL elongation, resulting in myopia (9, 10).

Studies have shown distinct AL growth patterns in myopic children, with accelerated AL growth in the year preceding myopia onset, followed by relatively slower and stable growth thereafter (11, 12). Rapid AL growth markedly increased the risk of axial myopia development, which is currently predominant in children and adolescents (13). This pattern is further supported by a large multicenter study involving 14,127 children and adolescents aged 4–18 years in China, which showed rapid AL growth between ages 4 and 9, slowing after age 9, and stabilizing around age 15 (14). Thus, in myopic children, if progression is not controlled, AL can continue to grow abnormally during this period, leading to high myopia (14).

Previous studies (15, 16) have consistently reported inverse correlations between AL and anterior segment parameters in both emmetropic and myopic populations. Longer AL was associated with flatter corneal curvature, decreased corneal thickness, and reduced endothelial cell density. However, one study (17) reported that this negative correlation between AL and corneal curvature disappeared when AL exceeded 28 mm. Furthermore, there remains no consensus on the relationship between AL and anterior segment parameters such as central corneal thickness (CCT) and lens thickness (LT), specifically in children and adolescents with high axial myopia. Thus, the main aim of this study was to understand the growth characteristics of AL and anterior segment biometry in children and adolescents with high axial myopia and to analyze their correlations.

## 2 Study population and methods

### 2.1 Study area and study population

The investigation was conducted in Chengdu City, China. According to the data published in the Seventh National Population Census of China (refer to Chengdu City’s Seventh National Population Census Report), as of 1 November 2020, the permanent population of Chengdu was 20.93 million. This study used a stratified cluster sampling method based on districts (counties) to select all primary and secondary schools from 8 administrative districts within 23 districts (counties) in Chengdu for investigation. Ultimately, 368,083 students from 916 schools (506 primary schools, 274 middle schools, and 136 high schools) in Chengdu were included. Following inclusion criteria and excluding individuals with incomplete information or those unwilling to undergo ophthalmic examinations, a final cross-sectional study of 366,278 students aged 7 to 18 years was established. The study commenced in January 2020 and concluded in December 2022.

### 2.2 Inclusion and exclusion criteria

Inclusion criteria included: (1) students enrolled in schools within Chengdu City (primary, middle, and high schools) and (2) an age range of 7 to 18 years.

Exclusion criteria included: (1) patients with various forms of glaucoma, corneal disorders, lens disorders, retinal disorders, and optic nerve diseases; (2) patients with amblyopia, strabismus, or severe visual function impairments; (3) patients with evident congenital or metabolic systemic diseases, such as trisomy 21 syndrome (Down syndrome, DS), type 1 diabetes, and similar conditions; (4) individuals with poor compliance, psychiatric disorders, or cognitive impairments; and (5) those who have worn corneal reshaping lenses or similar devices within the past 3 months.

### 2.3 Study design

This study was a large-scale cross-sectional survey based on school myopia screening, with children and adolescents as research subjects. Students in Chengdu undergo two eye examinations annually as part of routine myopia screening. In order to better implement myopia screening on the Chengdu campus, a dedicated myopia screening working group was established. During the peak months (March to April and September to October), up to 12 groups were divided, each group consisted of 1 ophthalmologist or PhD student with a medical license and 6 nurses. Each team was equipped with three light-box E-word standard logarithmic visual acuity charts (GB 11533), one Topcon Fully-Automated Kerato-Refractometer, and three SUOERμ-Meter optical biometric instruments. Each group screened an average of approximately 200 students per day. The project was officially initiated in January 2020. With the assistance of the Chengdu Education Bureau and Health Bureau, the Ineye Hospital of Chengdu University of Traditional Chinese Medicine (TCM) collected basic student information in advance, including school type, school name, grade, class, name, gender, and age. An eye health record was established through an eye health record system, encompassing all student information and a unique identification code. This identification code was utilized for transmitting examination results. All participants underwent ophthalmic examinations, including unaided visual acuity assessment, non-cycloplegic autorefraction, and ocular biometric parameters assessment.

### 2.4 Inspection methods

Non-cycloplegic visual acuity and refraction assessments were performed. Uncorrected visual acuity (UCVA) was measured for each eye using a standard logarithmic visual acuity chart at 5 meters under standard illumination and recorded in logMAR notation. Subsequently, autorefraction was conducted. Before the examination, screening personnel scanned the unique identification code of the student to initiate the process. Examination results were automatically uploaded to the eye health record system. The examination used the Topcon Fully-Automated Kerato-Refractometer (KR-1, Japan Topcon Healthcare Trading Co., Ltd.). The height of the KR-1 was set based on the height of the surveyed children and adolescents. Each eye was measured at least three times. If one measurement differed from the other two measurements by more than the maximum allowable error (spherical equivalent within the range of 0.50 to 0.75D), the measurement was repeated. The average of three reliable measurements was used for the final statistical analysis.

**Figure 1:**
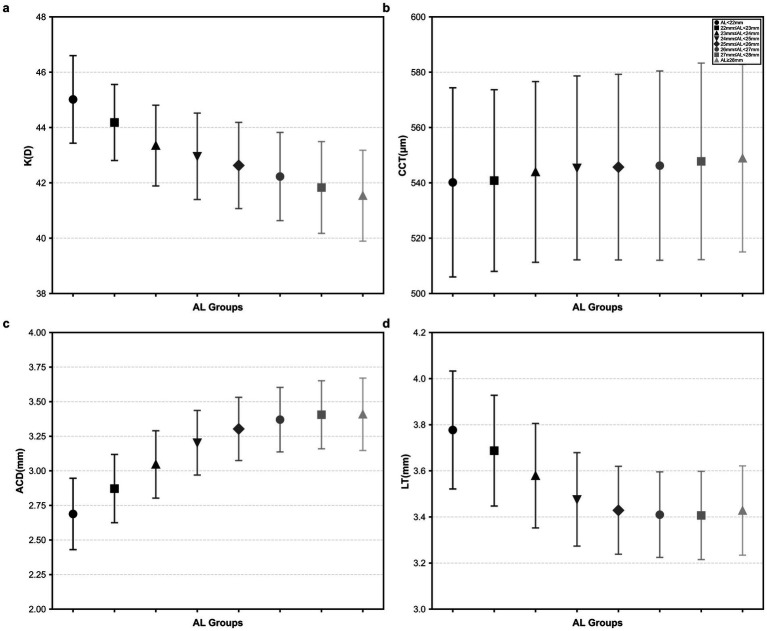
Anterior segment biometry among different AL groups. **(a)** Trends in the K among different AL groups; **(b)** Trends in the CCT among different AL groups; **(c)** Trends in the ACD among different AL groups; and **(d)** Trends in the LT among different AL groups (the points represent the mean, and error bars represent the standard deviation).

Measurement of ocular biometric parameters was performed using the SUOERμ-Meter optical biometric instrument (SW-9000, Tianjin Suoerwei Electronic Technology Co., Ltd.). To ensure data accuracy, the instrument was calibrated daily prior to data collection. The SUOERμ-Meter optical biometric instrument was used to measure the optic axis. The fast measurement speed and non-contact are intended for patient comfort. Capturing eight different measurements in less than 5 s, which are corneal thickness, anterior chamber depth, lens thickness, axial length, corneal curvature, axial angle, white-to-white distance measurement (corneal diameter), and pupil diameter. The average of three reliable measurements was used for the final statistical analysis. High axial myopia was defined as an AL ≥ 26 mm (17), super-high axial myopia was defined as an AL ≥ 28 mm. The following analyses were conducted using right eye statistics. Following the method outlined by Jin et al. (17), the AL was segmented into 1 mm intervals for analysis (Figure 1). This segmented approach aids in elucidating the finer relationship between AL development and anterior segment biometry, enhancing the precision of this study. It enables researchers to specifically investigate the complex relationship between AL and anterior segment biometry.

### 2.5 Statistical methods

All statistical analyses were performed using SPSS software (version 26.0, Chicago, IL, USA). Continuous variables that follow a normal distribution were expressed as mean ± standard deviation, while those that do not follow a normal distribution were described as median (25th percentile, 75th percentile). Comparisons of continuous variables were performed using independent samples *t*-test and one-way analysis of variance (ANOVA) for normally distributed variables, and Mann–Whitney *U*-test or Kruskal–Wallis test for non-normally distributed variables.

Pearson’s correlation analysis was used to assess correlations. The relationship between AL and anterior segment biometry was investigated using multiple linear regression models, adjusted for age and gender, as well as segmented multiple linear regression models. The significance level was set at *α* = 0.05. Given the high correlation (*r* = 0.96) between the AL of the left and right eyes, the analysis in this study focused primarily on the right eye.

### 2.6 Medical ethics

This study received approval from the Ethics Committee of Ineye Hospital of Chengdu University of TCM (2019yh-007). All research methods adhered to the principles outlined in the “Declaration of Helsinki.” Before conducting the study, the objectives and methods were presented to the principals, teachers, and parents of the participating schools to obtain informed consent.

## 3 Results

### 3.1 Study population

After a 3-year student eye health study conducted in Chengdu, China (January 2020–December 2022), data from 366,278 enrolled students were collected. This information included school type, school name, grade, class, name, gender, and age, along with refractive errors and various ocular biometric parameters. The data were obtained from 916 schools (506 primary schools, 274 middle schools, and 136 high schools), covering a population of children and adolescents aged 7–18 years. Among this group, 52.06% (190,674) were male, and 47.94% (175,604) were female.

### 3.2 Diopter and ocular biometric parameters

Significant age- and gender-related differences were observed across all measured parameters (SE, K, AL, CCT, ACD, LT, and UCVA; all *p* < 0.001). Female students exhibited significantly longer AL and deeper ACD compared to male students, whereas male students demonstrated steeper corneal curvature (K) and lens thickness (LT). Participants were classified into three refractive status groups: non-myopia (35.09%, *n* = 128,549), normal myopia with AL < 26 mm (57.02%, *n* = 208,852), and high axial myopia (7.89%, *n* = 28,877). All ocular parameters differed significantly among the three refractive status groups (all *p* < 0.001). The high axial myopia group exhibited the most pronounced characteristics with the longest AL, deepest ACD, thinnest LT, and poorest UCVA among all groups (Table 1).

**Table 1:** Distribution of the anterior segment biometry and axial length at different ages and genders.

Participant characteristics		*N*	SE(D)	K(D)	AL(mm)	CCT(um)	ACD(mm)	LT(mm)	UCVA(LogMAR)
Age	7	41,809	−0.12 ± 1.01	43.26 ± 1.59	23.03 ± 0.74	541.09 ± 32.09	2.91 ± 0.27	3.69 ± 0.25	0.06 ± 0.16
	8	36,454	−0.46 ± 1.22	43.27 ± 1.60	23.37 ± 0.82	542.88 ± 32.23	3.01 ± 0.28	3.60 ± 0.25	0.12 ± 0.24
	9	37,352	−0.83 ± 1.40	43.24 ± 1.58	23.68 ± 0.89	544.07 ± 32.47	3.08 ± 0.27	3.54 ± 0.23	0.19 ± 0.30
	10	34,594	−1.20 ± 1.58	43.21 ± 1.60	23.94 ± 0.95	544.46 ± 32.63	3.13 ± 0.27	3.50 ± 0.22	0.27 ± 0.34
	11	33,259	−1.57 ± 1.73	43.21 ± 1.60	24.16 ± 1.01	545.19 ± 32.85	3.16 ± 0.27	3.48 ± 0.22	0.33 ± 0.36
	12	34,860	−1.94 ± 1.89	43.19 ± 1.61	24.38 ± 1.06	545.89 ± 33.17	3.18 ± 0.27	3.48 ± 0.21	0.40 ± 0.38
	13	32,499	−2.39 ± 2.02	43.15 ± 1.61	24.59 ± 1.10	546.04 ± 33.42	3.21 ± 0.27	3.48 ± 0.21	0.48 ± 0.39
	14	30,998	−2.77 ± 2.14	43.17 ± 1.61	24.76 ± 1.15	545.96 ± 33.22	3.21 ± 0.27	3.48 ± 0.21	0.55 ± 0.39
	15	30,782	−2.97 ± 2.20	43.12 ± 1.62	24.86 ± 1.19	545.21 ± 33.82	3.21 ± 0.27	3.50 ± 0.21	0.59 ± 0.40
	16	24,528	−3.07 ± 2.28	43.03 ± 1.75	24.90 ± 1.23	544.20 ± 34.69	3.20 ± 0.27	3.52 ± 0.21	0.61 ± 0.40
	17	21,027	−3.30 ± 2.38	43.03 ± 1.78	25.01 ± 1.25	543.66 ± 35.36	3.20 ± 0.28	3.53 ± 0.22	0.63 ± 0.40
	18	8,116	−3.42 ± 2.44	43.17 ± 1.78	25.02 ± 1.29	543.11 ± 36.05	3.18 ± 0.28	3.55 ± 0.22	0.62 ± 0.40
	All	366,278	−1.75 ± 2.10	43.18 ± 1.63	24.17 ± 1.22	544.32 ± 33.22	3.13 ± 0.29	3.53 ± 0.23	0.36 ± 0.39
	*p*-value	–	<0.001	<0.001	<0.001	<0.001	<0.001	<0.001	<0.001
Gender	Male	190,674	−1.68 ± 2.11	42.85 ± 1.59	24.41 ± 1.21	546.00 ± 33.26	3.18 ± 0.29	3.51 ± 0.23	0.34 ± 0.39
	Female	175,604	−1.83 ± 2.09	43.55 ± 1.59	23.91 ± 1.17	542.50 ± 33.09	3.07 ± 0.28	3.55 ± 0.24	0.39 ± 0.40
	*p-*value	–	<0.001	<0.001	<0.001	<0.001	<0.001	<0.001	<0.001
Refractive status	NM^a^	128,549	0.20 ± 0.58	43.11 ± 1.60	23.28 ± 0.77	544.22 ± 33.10	2.98 ± 0.27	3.62 ± 0.24	0.04 ± 0.14
	M^b^	208,852	−2.46 ± 1.53	43.37 ± 1.59	24.38 ± 0.90	544.06 ± 33.11	3.18 ± 0.26	3.50 ± 0.22	0.49 ± 0.37
	HAM^c^	28,877	−5.32 ± 2.07	42.13 ± 1.62	26.64 ± 0.58	546.60 ± 34.44	3.38 ± 0.24	3.41 ± 0.19	0.84 ± 0.28
	*p*-value	–	<0.001	<0.001	<0.001	<0.001	<0.001	<0.001	<0.001

ᵃ NM, non-myopia group (SE ≥ − 0.05); ^b^ M, myopia group (SE <−0.05 and AL <26 mm); ^c^ HAM, high axial myopia group (AL ≥ 26 mm); SE, spherical equivalent; AL, axial length; K, average keratometry; CCT, central corneal thickness; ACD, anterior chamber depth; LT, lens thickness; UCVA, uncorrected visual acuity.

### 3.3 Comparison of anterior segment parameters across refractive status groups

**Figure 2:**
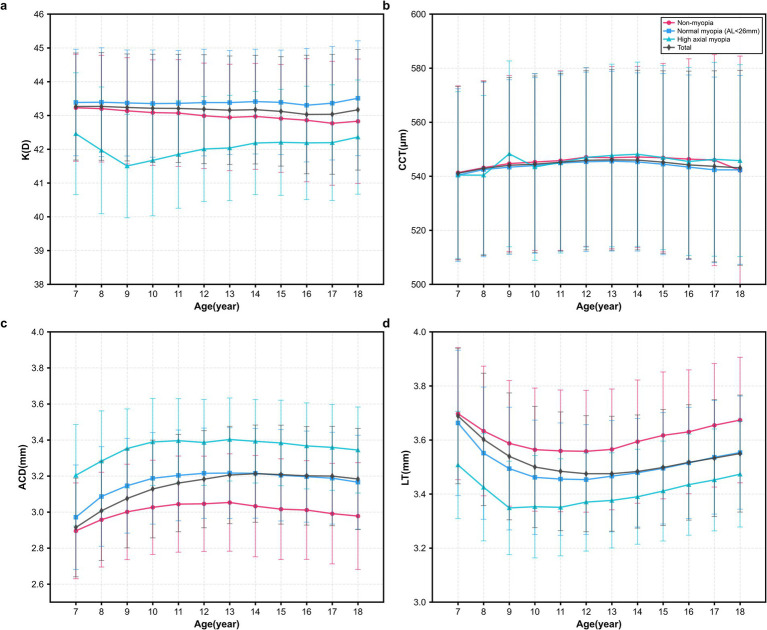
Anterior segment biometry among different age groups. **(a)** Trends in the K among different age groups; **(b)** Trends in the CCT among different age groups; **(c)** Trends in the ACD among different age groups; and **(d)** Trends in the LT among different age groups. (The points represent the mean, and error bars represent the standard deviation).

Cross-sectional analysis comparing the three refractive status groups revealed significant differences in anterior segment parameters, independent of age and gender effects. The high axial myopia group exhibited significantly higher K values, thicker CCT, deeper ACD, and lower LT compared to both the non-myopia and normal myopia groups (all *p* < 0.001). The normal myopia group demonstrated intermediate values for these parameters, showing significant differences from the non-myopia group (all *p* < 0.05) but less pronounced changes than the high axial myopia group (Table 2; Figure 2).

**Table 2:** Comparison of ocular biometric parameters among different refractive status groups by age and gender.

Participant characteristics		K(D)			CCT(um)			ACD(mm)			LT(mm)		
		NM	M	HAM	NM	M	HAM	NM	M	HAM	NM	M	HAM
Age	7	43.23 ± 1.59	43.38 ± 1.58*	42.46 ± 1.80†	541.32 ± 32.13	540.39 ± 31.98*	540.44 ± 30.85	2.90 ± 0.27	2.97 ± 0.29*	3.20 ± 0.28†	3.70 ± 0.24	3.66 ± 0.27*	3.51 ± 0.20†
	8	43.20 ± 1.58	43.39 ± 1.61*	41.97 ± 1.88†	543.14 ± 32.22	542.47 ± 32.26	540.40 ± 29.48	2.96 ± 0.26	3.09 ± 0.28*	3.28 ± 0.28†	3.63 ± 0.24	3.55 ± 0.24*	3.42 ± 0.20†
	9	43.14 ± 1.57	43.37 ± 1.57*	41.50 ± 1.53†	544.68 ± 32.55	543.36 ± 32.33*	548.30 ± 34.39†	3.00 ± 0.26	3.15 ± 0.26*	3.35 ± 0.22†	3.59 ± 0.23	3.49 ± 0.23*	3.35 ± 0.17†
	10	43.08 ± 1.56	43.35 ± 1.59*	41.67 ± 1.64†	545.25 ± 32.71	543.97 ± 32.51*	543.44 ± 34.54	3.03 ± 0.26	3.19 ± 0.25*	3.39 ± 0.24†	3.56 ± 0.23	3.46 ± 0.21*	3.35 ± 0.19†
	11	43.07 ± 1.58	43.36 ± 1.56*	41.85 ± 1.60†	545.79 ± 33.23	544.90 ± 32.62*	545.13 ± 33.55	3.04 ± 0.27	3.20 ± 0.25*	3.40 ± 0.23†	3.56 ± 0.23	3.46 ± 0.21*	3.35 ± 0.18†
	12	42.99 ± 1.56	43.38 ± 1.58*	42.01 ± 1.55†	547.05 ± 33.07	545.34 ± 33.19*	546.97 ± 33.17#	3.05 ± 0.26	3.22 ± 0.25*	3.39 ± 0.24†	3.56 ± 0.23	3.45 ± 0.20*	3.37 ± 0.18†
	13	42.94 ± 1.57	43.38 ± 1.54*	42.04 ± 1.56†	546.87 ± 33.71	545.57 ± 33.27*	547.68 ± 33.77#	3.05 ± 0.27	3.22 ± 0.25*	3.40 ± 0.23†	3.57 ± 0.22	3.47 ± 0.21*	3.38 ± 0.18†
	14	42.97 ± 1.57	43.41 ± 1.55*	42.18 ± 1.53†	547.13 ± 33.51	545.28 ± 32.96*	548.14 ± 34.11#	3.03 ± 0.28	3.22 ± 0.25*	3.39 ± 0.23†	3.59 ± 0.23	3.48 ± 0.20*	3.39 ± 0.18†
	15	42.91 ± 1.60	43.39 ± 1.55*	42.20 ± 1.57†	546.86 ± 34.87	544.45 ± 33.51*	546.90 ± 34.06#	3.02 ± 0.28	3.20 ± 0.25*	3.38 ± 0.24†	3.62 ± 0.23	3.50 ± 0.21*	3.41 ± 0.18†
	16	42.86 ± 1.82	43.30 ± 1.68*	42.19 ± 1.68†	546.35 ± 37.13	543.38 ± 34.08*	545.46 ± 34.77#	3.01 ± 0.27	3.20 ± 0.25*	3.37 ± 0.24†	3.63 ± 0.23	3.52 ± 0.21*	3.43 ± 0.19†
	17	42.77 ± 1.83	43.36 ± 1.68*	42.19 ± 1.71†	545.96 ± 39.05	542.36 ± 34.35*	546.26 ± 35.87#	2.99 ± 0.28	3.19 ± 0.25*	3.36 ± 0.24†	3.65 ± 0.23	3.54 ± 0.21*	3.45 ± 0.19†
	18	42.83 ± 1.84	43.51 ± 1.70*	42.36 ± 1.69†	542.16 ± 42.29	542.36 ± 34.94	545.77 ± 35.50†	2.98 ± 0.30	3.17 ± 0.26*	3.34 ± 0.24†	3.67 ± 0.23	3.55 ± 0.21*	3.47 ± 0.20†
Gender	Male	42.79 ± 1.56	43.06 ± 1.56*	42.01 ± 1.58†	545.86 ± 33.00	545.79 ± 33.28	547.48 ± 33.98†	3.04 ± 0.27	3.23 ± 0.26*	3.41 ± 0.23†	3.60 ± 0.24	3.48 ± 0.22*	3.40 ± 0.19†
	Female	43.49 ± 1.56	43.66 ± 1.56*	42.43 ± 1.67†	542.23 ± 33.12	542.50 ± 32.88	544.42 ± 35.47†	2.91 ± 0.27	3.13 ± 0.26*	3.31 ± 0.23†	3.65 ± 0.24	3.51 ± 0.22*	3.43 ± 0.19†

NM, non-myopia group (SE ≥ − 0.05); M, myopia group (SE < −0.05, and AL <26 mm); HAM: high axial myopia group (AL ≥ 26 mm).

* means *p* < 0.05 vs. NM group; † means *p* < 0.05 vs. both non-myopia and normal myopia groups.

K, average keratometry; CCT, central corneal thickness; ACD, anterior chamber depth; LT, lens thickness.

### 3.4 Correlation between anterior segment biometry and AL

Pearson’s correlation analysis revealed significant associations between AL and all anterior segment parameters (all *p* < 0.001). AL exhibited moderate negative correlations with both K and LT, a strong positive correlation with ACD, and a weak but significant positive correlation with CCT.

Inter-parameter correlations among anterior segment biometric parameters were also examined. ACD and LT demonstrated a moderate negative correlation (*p* < 0.001). K showed a weak negative correlation with both CCT and LT (both *p* < 0.001), while demonstrating a negligible correlation with ACD (*p* = 0.006). Weak but significant correlations were observed between CCT and both ACD and LT (both *p* < 0.001) (Table 3).

**Table 3:** Pearson’s correlation coefficients among ocular parameters.

Variable	*r*	95% CI	*p*-value
AL vs. K	−0.3746	[−0.3774, −0.3718]	<0.001
AL vs. CCT	0.0507	[0.0475, 0.054]	<0.001
AL vs. ACD	0.5671	[0.5649, 0.5693]	<0.001
AL vs. LT	−0.3968	[−0.3996, −0.3941]	<0.001
K vs. CCT	−0.1184	[−0.1216, −0.1152]	<0.001
K vs. ACD	0.0045	[0.0013, 0.0078]	0.005912
K vs. LT	−0.0494	[−0.0527, −0.0462]	<0.001
CCT vs. ACD	−0.0735	[−0.0767, −0.0703]	<0.001
CCT vs. LT	0.0254	[0.0221, 0.0286]	<0.001
ACD vs. LT	−0.5963	[−0.5984, −0.5942]	<0.001

AL, axial length; K, average keratometry; CCT, central corneal thickness; ACD, anterior chamber depth; LT, lens thickness.

### 3.5 Multiple linear regression analysis with AL

The multiple linear regression model was constructed to estimate the AL based on age, gender, and anterior segment biometry, yielding an R-squared value of 0.601 (*F* = 91948.012, *p* < 0.001). After adjusting for gender and age, K, CCT, ACD, and LT were significantly associated with AL (for K, *β* = −0.265 [95%CI −0.267 to −0.264]; for CCT, *β* = 0.001 [95%CI 0.001 to 0.001]; for ACD, *β* = 1.609 [95%CI 1.598 to 1.620]; for LT, *β* = −0.607 [95%CI −0.620 to −0.594]; all *p*-values<0.001). However, after adjusting for gender and age, LT was significantly negatively associated with AL only when AL was <26 mm (*β* = −1.379 [95%CI −1.390 to −1.367], *p* < 0.001) and when AL was between 26 and 27 mm (*β* = −0.027 [95%CI −0.047 to −0.008], *p* < 0.01). There was no significant association between LT and AL when AL was between 27 and 28 mm (*β* = 0.025 [95%CI −0.013 to 0.064], *p* = 0.195), and when AL was ≥28 mm (*β* = 0.043 [95%CI −0.111 to 0.198], *p* = 0.581). After adjusting for gender and age, CCT was significantly negatively associated with AL only when AL was <26 mm (*β* = 0.002 [95%CI 0.001to 0.002], *p* < 0.001). There was no significant association between CCT and AL when AL was between 26 and 27 mm (*β* = −3.787E-5 [95%CI 0.000 to 0.000], *p* = 0.497), when between 27 and 28 mm (*β* = 1.134E-5 [95%CI 0.000 to 0.000], *p* = 0.914), and when AL was ≥28 mm (*β* = 0.000 [95%CI −0.001 to 0.000], *p* = 0.405) (Table 4).

**Table 4:** Multiple linear regression analysis with AL.

Parameters	*β*	95% CI	*p*-value
Age	0.142	0.141 to 0.143	<0.001
Gender	0.105	0.100 to 0.110	<0.001
K	−0.265	−0.267 to −0.264	<0.001
CCT	0.001	0.001 to 0.001	<0.001
ACD	1.609	1.598 to 1.620	<0.001
LT	−0.607	−0.620 to −0.594	<0.001

Age per year older; gender: male vs female; AL: axial length, per 1 mm longer; K, average keratometry, per 1D increase; CCT, central corneal thickness: per 1 μm increase; ACD, anterior chamber depth; per 1 mm longer; LT, lens per 1 mm longer.

## 4 Discussion

In this study, the anterior segment biometry was investigated in Chinese children and adolescents across three refractive status groups: non-myopia, normal myopia (AL < 26 mm), and high axial myopia (AL ≥ 26 mm). We analyzed the associations between AL and anterior segment biometry in children of similar for age and gender. The results indicated that the average K and LT in the high axial myopia group were significantly lower than those in both the non-myopia and normal myopia groups (all *p* < 0.001), whereas CCT and ACD were significantly greater in the high axial myopia group compared to the other two groups (all *p* < 0.001). Progressive trends were observed across the three groups, with normal myopia showing intermediate characteristics. As AL increased, CCT and ACD exhibited a growth trend, while K exhibited a decreasing trend. When AL was less than 27 mm, LT showed a thinning trend as AL increased. Similarly, when AL exceeded 27 mm, LT exhibited a gradual thickening trend with increasing AL. An AL of 27 mm served as the truncation point for the distribution of LT. Beyond an AL of 27 mm, there was no linear correlation between AL and LT, suggesting that the crystalline lens may gradually disappear as a compensatory mechanism for myopia.

This study revealed a negative correlation between AL and LT (*r* = −0.4, *p* < 0.001). As AL increases, LT exhibits a U-shaped curve trend. After AL surpasses 27 mm, LT gradually becomes thinner and then slowly thickens. Additionally, it was observed that there was no significant linear correlation between AL and LT. Previous research has also been conducted on 459 high myopic students aged 4 to 19 years, indicating that within a certain range of AL growth, changes in LP could potentially compensate for the effects of AL growth on myopia. However, this compensatory effect of the crystalline lens disappears when AL exceeds 27 mm (18). Related studies found that a sudden loss of compensatory changes in the crystalline lens characterizes the onset of myopia. Subsequently, the compensatory role of the crystalline lens weakened during the progression of myopia (19–21). Research also suggested that the rate of AL growth may be compensated for by an increased rate of LP loss, which continued until the year prior to the onset of myopia (22). For children and adolescents, the refractive state was primarily determined by the dynamic balance between AL and LP. Thinning of the lens thickness or reaching a certain physiological limit in LP loss can lead to a sudden decrease in LT or LP loss rate, accompanied by rapid AL growth, resulting in myopia (19, 23). Following the onset of myopia, as AL increases to a critical point, the compensatory ability of the lens may be diminished due to mechanisms such as abnormal thickening and elongation of the ciliary muscle, causing equatorial growth restrictions (24, 25).

This study also discovered that with increasing age, the LT becomes thinner until age 11, after which it gradually thickens. Moreover, the LT changes in individuals with high axial myopia were more pronounced than in both the non-myopia and normal myopia groups, consistent with findings from other studies on ocular growth (26). We speculated that apart from naturally thinning in children and adolescents with age (23), the thinning of the crystalline lens may play a role in compensating for myopia progression in axial elongation. However, the compensatory ability of the crystalline lens for myopia-related AL growth was limited. In the future, there should be a stronger emphasis on longitudinal cohort studies focusing on ocular biometry, especially in cases of myopia and high myopia, including observations of the ciliary muscle, to confirm the causal relationship between AL and LT.

The AL was the sum of corneal thickness, ACD, LT, and vitreous chamber depth. ACD was the distance from the inner surface of the cornea to the anterior surface of the lens, and studies have shown that changes in ACD were inversely proportional to lens thickness (26). Research indicated that ACD in young individuals increases with changes in refractive error, gradually deepening from the hyperopic to the high myopic group (27). Data also showed that eyes with longer AL (and more myopia) have deeper ACD and thinner LT, with myopic patients exhibiting a deeper ACD likely due to geometric scaling during AL growth (28). These findings were consistent with the results of our analysis. AL was positively correlated with ACD (*r* = 0.57, *p* < 0.001), ACD was negatively correlated with LT (*r* = −0.596, *p* < 0.001), and as AL increases, ACD gradually deepens, but the deepening trend of ACD became more gradual after AL exceeded 27 mm. The relationship between ACD and AL was influenced by ocular structure and physiological factors. When AL surpasses a certain threshold, LT no longer continues to thin and may even undergo compensatory thickening to maintain optical balance. This compensatory change in LT may help slow the rate of the ACD increase.

Earlier studies (15, 29, 30) have explored the negative correlation between corneal curvature and AL in the progression of myopia within an AL range of <28 mm. This suggested that the cornea compensates for AL growth through changes in its curvature, serving as a physiological adaptation for achieving optimal focusing. Similarly, in this study, it was observed that among different AL groups, high axial myopic children with longer AL have flatter corneas. Furthermore, it was noted that as AL continues to increase, the trend of corneal curvature changes slows down with increasing AL.

Wang et al. (31) have also found that as AL increases, corneal curvature becomes flatter to compensate for longer AL. However, this corneal compensation phenomenon disappeared when AL is >28 mm, as confirmed by Jin G (17). Similarly, in a long-term clinical cohort study involving myopic children and adolescents aged 7–18 over 14 years, a strong correlation was observed between corneal curvature and AL during the early stages of myopia progression (32). However, as myopia progression slowed and eventually stabilized, this correlation gradually diminished.

Additionally, in this study, it was observed that corneal curvature was significantly higher in females than in males of the same age, while AL was significantly shorter in females compared to males of the same age. This observation aligned with the results of a large-scale retrospective study conducted in Italy and was attributed to physiological differences between genders (33).

This study indicated that in the population of children and adolescents aged 7–18 years, CCT generally exhibited a slow thickening trend with increasing AL. However, when AL exceeded 26 mm, there was no linear correlation between AL and CCT (*p* > 0.05). This may be because corneal hysteresis was negatively correlated with axial elongation in children (34), and corneal hysteresis was statistically associated with CCT (35). A previous study involving 450 myopic individuals aged 21–66 years found that CCT was unrelated to AL, age, gender, or refractive error (36). In a study conducted in Hong Kong with 151 participants aged 10–60 years, CCT was observed to thin with increasing age, particularly in the age group of 10–25 years, where the decline in CCT was most significant (37). Other research has shown that CCT in the myopic group aged 18–55 years (527.7 ± 35.0 μm) was thinner compared to the emmetropic group (538.6 ± 32.1 μm) (38). Furthermore, a study involving participants with an average age of 22.2 ± 4.2 years revealed that CCT was thinner in cases of high myopia (16).

CCT may be correlated with body mass index (BMI) (39). Conversely, thinning of CCT in high myopia may be due to the rapid progression of myopia, resulting in rapid AL growth, scleral thinning, and potential thinning of the corneal stroma (40). Additionally, Zhou P (41) found that CCT was negatively correlated with the rate of myopia progression (spherical equivalent, SE) and AL growth rate. Children with thinner CCT tend to have faster myopia progression and AL growth rates. Current research has generated controversy regarding the relationship between CCT and AL at different age stages. The main point of contention revolved around the physiological thickening of CCT during the rapid growth and development period in children and adolescents, as opposed to the pathological thinning of CCT due to continuous axial elongation. In light of these findings, CCT may be considered a potential risk factor for myopia, or it may be a consequence of myopia development. Further research is required to validate these findings, which could contribute to the development of more effective strategies for myopia prevention and treatment.

This study demonstrated significant gender-related differences in anterior segment biometry, with males exhibiting longer AL, deeper ACD, and flatter corneas, whereas females showed thicker crystalline lenses (all *p* < 0.001). These findings were consistent across pediatric (42, 43) and adult cohorts (15, 33), suggesting early establishment and persistence of gender dimorphism in ocular development. Such coordinated gender-specific patterns suggest compensatory mechanisms for maintaining optical balance during emmetropization (42, 43). These differences have important implications for refractive development prediction and personalized myopia management.

This study included a student population in Chengdu, aged 7–18 years, totaling 366,278 individuals. This extensive sample size improved the credibility and reliability of the study results, making them more representative and generalizable. Additionally, the study conducted comprehensive measurements of ocular biometric parameters in 366,278 children and adolescents, including AL, K, LT, ACD, and CCT, among others. This study provided a comprehensive dataset on ocular structure and ocular biometric parameters, thereby enriching the field of research. However, several limitations should be noted in this study. The cross-sectional design includes inference of causal relationships for associations observed in different age groups, especially regarding the relationship between LT and AL. To address these limitations, our research team plans to strengthen longitudinal cohort studies in future research, allowing for a more precise and scientific analysis of myopia-related data.

In summary, findings from this large-scale study of Chinese children and adolescents aged 7–18 years indicated that, in high axial myopic patients, the average K and LT were significantly lower than both the non-myopia and normal myopia groups, while CCT and ACD were significantly greater than the other two groups. Within an AL range of 27 mm, changes in crystalline lens shape provide optical compensation for myopia. However, in cases of extremely high myopia (AL > 27 mm), this effect was not detected. Furthermore, beyond an AL of 26 mm, there was no linear correlation observed between AL and CCT. As AL increases, pathological thinning of CCT gradually surpasses physiological thickening. In the high myopic population studied, there was limited evidence of regulatory changes in the LT and CCT associated with AL growth. These findings highlight the importance of focusing on AL growth characteristics and anterior segment biometry in children and adolescents with high myopia. Such an approach will enable a more accurate use of ocular biometric parameters for assessing and predicting high myopia, thereby aiding in the early detection and prevention of potential complications.

## Data Availability

The datasets presented in this article are not readily available because the datasets used and/or analysed during the current study available from the corresponding author on reasonable request. Requests to access the datasets should be directed to Junguo Duan, zeverett636@hotmail.com.
